# Brain Disposition of Antibody-Based Therapeutics: Dogma, Approaches and Perspectives

**DOI:** 10.3390/ijms22126442

**Published:** 2021-06-16

**Authors:** Aida Kouhi, Vyshnavi Pachipulusu, Talya Kapenstein, Peisheng Hu, Alan L. Epstein, Leslie A. Khawli

**Affiliations:** Department of Pathology, Keck School of Medicine of University of Southern California, Los Angeles, CA 90033, USA; kouhi@usc.edu (A.K.); pachipul@usc.edu (V.P.); talya.kapenstein@gmail.com (T.K.); peisheng@usc.edu (P.H.); aepstein@usc.edu (A.L.E.)

**Keywords:** blood–brain barrier, antibody, pharmacokinetics, disposition, biochemical and physicochemical properties, Fc binding, receptor-mediated transcytosis, brain shuttle, molecular Trojan horse, transferrin

## Abstract

Due to their high specificity, monoclonal antibodies have been widely investigated for their application in drug delivery to the central nervous system (CNS) for the treatment of neurological diseases such as stroke, Alzheimer’s, and Parkinson’s disease. Research in the past few decades has revealed that one of the biggest challenges in the development of antibodies for drug delivery to the CNS is the presence of blood–brain barrier (BBB), which acts to restrict drug delivery and contributes to the limited uptake (0.1–0.2% of injected dose) of circulating antibodies into the brain. This article reviews the various methods currently used for antibody delivery to the CNS at the preclinical stage of development and the underlying mechanisms of BBB penetration. It also describes efforts to improve or modulate the physicochemical and biochemical properties of antibodies (e.g., charge, Fc receptor binding affinity, and target affinity), to adapt their pharmacokinetics (PK), and to influence their distribution and disposition into the brain. Finally, a distinction is made between approaches that seek to modify BBB permeability and those that use a physiological approach or antibody engineering to increase uptake in the CNS. Although there are currently inherent difficulties in developing safe and efficacious antibodies that will cross the BBB, the future prospects of brain-targeted delivery of antibody-based agents are believed to be excellent.

## 1. Introduction

Drug uptake into the brain is quite challenging, although not impossible [1]. Since the brain is located in a non-expandable vault (cranium) and is very sensitive to pressure and the environment, cerebrospinal fluid (CSF) flow in and out of the brain is highly regulated and controls the selective uptake of key nutrients and fluid to maintain normal brain function. This regulation also includes the passage of large molecules such as immunoglobulin thereby accounting for the observed difficulties of targeting the central nervous system (CNS) with therapeutic proteins and reagents. Many potentially useful drugs, which, because of their low entrance into the CNS, are not being used to treat brain disease. This lack of access to the brain has been described as a major hurdle in the development of large biomolecules and a reason given for their comparatively long development times and high failure rate [2]. As a consequence, several approaches are currently being investigated to enhance the CNS delivery of various types of large biomolecules, such as antibodies, recombinant proteins, gene vectors, liposomes, and nanoparticles (Table 1). To evaluate CNS delivery, quantitative measurements are used to understand better and potentially even improve upon methods for the targeted delivery of antibody-based therapeutics across the BBB. In particular, scientific and technological advancements that focus on evaluating methods for altering antibody penetration and distribution in the brain have not yet been developed adequately to treat neurological diseases. Moreover, even if candidate antibodies for the therapy of CNS diseases may be already available, they cannot currently be utilized because of their poor blood-to-brain penetration due to the presence of the tight-junctioned BBB preventing the passage of antibodies [3]. Thus, increased attention is being placed on novel antibodies capable of successfully enhancing brain tissue concentration as well as targeting specific disease regions within the CNS [4,5]. If proven safe and effective, these new technologies could represent the future of antibody therapy in the treatment of neurologic diseases.

Here, we review some of the most important principles and multiple strategies for enhancing antibody delivery to the brain and discuss how they can be applied to the pre-clinical development of CNS therapeutics. The guiding principles and knowledge gained from preclinical evaluation of these different strategies for CNS-targeting antibodies that are currently under development are also discussed, with a particular emphasis on pharmacokinetic (PK) and disposition properties. In addition, this review includes a brief description of the physicochemical and biochemical interactions between antibodies and biological matrices. As such, focus is given to defining the general properties of antibodies, their similarities and differences with regard to charge, neonatal Fc receptor (FcRn) binding, and target affinity. These types of studies provide scientists with the knowledge necessary to select the appropriate antibody characteristics to maximize brain exposure, which in turn, could provide better efficacy of their product. Finally, an improved understanding of the effects of these critical characteristics may allow for the better design of novel antibody therapeutics with unique and useful properties that conceptually are able to efficiently cross the BBB [5]. Hence, the objective of this review was to describe the progress of antibody-based drugs and highlight the principles and existing approaches for enhancing their entrance into the brain to achieve a desirable concentration range for the therapy of CNS disease.

## 2. Delivery of Antibodies into the Brain: Mechanism of Delivery 

Diseases of the CNS are in need of effective biotherapeutics. However, the CNS has been considered off-limits to antibody therapeutics because of the presence of the BBB, which separates the circulating blood from the brain and extracellular fluid in the CNS to prevent brain uptake of most large molecules [11,15]. Recent advances in preclinical and clinical drug development suggest that antibodies can cross the BBB in limited quantities and act centrally to mediate their effects [4]. In particular, immunotherapy studies of AD have shown that targeting beta amyloid with antibodies can reduce disease pathology in both mouse models and patients, with strong evidence supporting a central mechanism of action.

### 2.1. Physiology and Barriers of the CNS 

The arrangement of cells at the interface between the blood and the CNS restricts both the paracellular and transcellular diffusion of hydrophilic and hydrophobic substances into the CNS [16]. The blood–brain barrier (BBB) is used to describe the barrier between the blood and the brain and spinal cord parenchyma proper. At this interface, cerebral microvessels, lined with endothelial cells, limit the passage of small molecules from the blood into the brain or spinal cord [11]. Microvascular endothelial cells make up a large portion of the brain’s surface area, which helps account for its ability to restrict the flow of substances into the brain [17]. A second barrier, referred to as the blood–CSF barrier, exists between the blood and the ventricular CSF. Formed by CSF producing tight-junctioned epithelium of the choroid plexuses, this epithelial cell barrier accounts for a significant surface area of exchange [16]. Additionally, the blood flow rate within the choroid plexuses is higher than any other brain structures, and therefore, the blood flow through these areas significantly contributes to exchanges between the blood and the CNS. A third barrier to the CNS is the arachoid membrane, which completely encircles the CNS and separates the subarachnoid CSF from the bones and *dura mater* extracellular fluids [16,18]. These three barriers to the CNS work to manage the traffic of small and large molecules from the blood into the brain.

### 2.2. BBB Structure 

As described above, the BBB consists of the network of cells that communicate and associate together to form a barrier between the interstitial fluid of the brain and circulating blood. A thin monolayer of brain microvascular endothelial cells (BMECs) joined together by tight junction forms the physical BBB. The BMECs are supported by the capillary basement membrane, pericytes, astrocytes, and microglial cells. It is the interaction of the BMECs with these other cell types that creates the specific brain microvascular network. The tight junctions are responsible for the selective permeability of the BBB, as they seal the apical region of the endothelial cells together and restrict the entrance of hydrophilic drugs into the brain. Additionally, actin filaments, such as cadherins and catenins, arranged below the tight junctions, link together to form a band of adherence junctions. These adherence junctions contribute to the brain barrier, and also, among other roles, they promote BMECs adhesions, cell polarity, and control paracellular permeability regulations. It is the dynamic interaction between the tight junctions and the adhesion junctions and the other cellular components of the BBB via signaling pathways that regulate the BBB’s permeability. The arrangement of cells that form the BBB allow it to have uniform thickness, a negative surface charge, little pinocytotic activity, and no fenestrae [19].

### 2.3. Pharmacokinetics and CNS Distribution of Antibodies 

The PK properties of therapeutic antibodies are an essential factor that determine their in vivo efficacy by impacting their biodistribution and have been extensively studied in recent years [20]. The processes that govern the biodistribution of therapeutic antibodies depends on the species they are administered to and on the properties of the antibody itself. While physiological conditions are frequently constant, various properties of a therapeutic antibody such as its charge or size can be modified during development in order to optimize its PK behavior. Structural modifications such as glycosylation can also impact the biodistribution of an antibody. Of particular importance, however, is the role of FcRn on PK properties of an antibody, which must be considered in designing therapeutic antibodies for neurological disorders. FcRn is a receptor that is highly expressed in various tissues and prolongs an IgG antibody’s half-life by protecting it from lysosomal degradation. It has been reported that the receptor contributes to the efflux of IgG therapeutic antibodies at the BBB and can reduce brain uptake following administration despite prolonging its half-life. The crucial role of FcRn on the CNS distribution behavior of antibodies is further discussed in Section 4. 

### 2.4. Mechanisms of Antibody Passage Across the BBB

In the past few decades, various transport mechanisms have been identified as major pathways for macromolecules to cross the BBB. Generally, approximately 0.1% of circulating antibodies enter the brain. Mechanisms in play include: i) Adsorptive-mediated endocytosis; (ii) Carrier-mediated transport; and (iii) Receptor-mediated transcytosis.

(i) Adsorptive-mediated endocytosis (AMT) is a mechanism of BBB transport that relies on an electrostatic interaction between a cationic molecule in the circulation and the negatively charged cell membrane at the BBB, which will in turn trigger internalization of the positively charged molecule [3]. Cationic modification of proteins such as albumin and IgGs have been used to enhance their uptake into the brain. Studies have demonstrated that cationization of antibodies by covalently linking primary amine groups to their surface enhances their uptake into the brain by AMT. The capacity of AMT is high, but this mechanism is low in affinity and therefore has poor specificity. This is because cationized molecules can interact with negatively charged cell membranes of peripheral organs so that uptake in the brain does not increase proportionally [4,21]. The non-specificity of AMT mechanism should be considered in designing therapeutic antibodies that are targeted to the brain [6]. 

(ii) Carrier-mediated transport (CMT) is a mechanism by which small molecules such as glucose, amino acids, vitamins, hormones, and other nutrients rapidly cross the BBB [4]. This is a saturable mechanism due to the engagement of carriers and maintains homeostasis in the CNS by transporting these molecules bidirectionally [3]. Carrier-mediated transporters include CLUT1, which mediates transport of glucose and mannose and LTA1, which mediates transport of large neutral amino acids [21]. In principle, molecules can enter the brain using the CMT if they are conjugated to either endogenous ligands of the carriers or their analogues. However, this process has proved to be challenging for transport of antibodies because these carriers transport small molecules and are highly stereoselective. 

(iii) Receptor-mediated transcytosis (RMT) is one of the most promising approaches for delivering antibodies to the brain [4]. There are three categories of receptors that mediate RMT: iron transporters such as transferrin receptors (TfR); insulin transporters such as insulin receptor (IR); and lipid transporters such as low-density lipoprotein receptor- related protein 1 (LRP1). This process entails binding of the ligand to the receptor, internalization of the ligand–receptor complex, and exocytosis on the abluminal side of the cell [3]. It is important to keep in mind, however, that high-affinity antibodies toward receptors that mediate RMT will follow the lysosomal pathway when internalized, which results in their degradation [22]. While this phenomenon creates a challenge in using the RMT mechanism, optimizing the affinity of the ligand that is targeting these receptors has proved to be an effective strategy [22]. 

## 3. Current In Vitro and In Vivo Methodologies for Measuring Brain Access of Antibodies: Advantages and Limitations

Implementation of in vitro models of the BBB that correlate with in vivo studies would provide desirable preclinical tools for the mechanistic understanding of drug transport via brain endothelial cells and uptake into the CNS monitored by the BBB. Use of these as a screening tool are of critical importance for the determination of drug permeability, PK, and distribution to brain tissues and cells.

### 3.1. In Vitro Methods

To aid in our understanding of the role of the BBB in protecting the brain microenvironment, different types of in vitro models of the BBB have been developed, which are classified into either static or dynamic BBB models [19,23]. Static BBB models are commonly used, but they do not imitate the shear stress, which is usually generated in vivo due to the blood flow. Static BBB models are further divided into monolayer and co-culture models, based on type of cells involved in the BBB design. While the brain microvessel endothelial cell culture model presents many differences compared with the in vivo system, monolayer cultures in a trans-well system allow a simple method for drug screening and permeability studies. The co-culture BBB model, however, is used to mimic the anatomic structure of BBB in vivo, in which BMECs are co-cultured with other CNS cells that directly contribute to the barrier properties of the BBB. As none of these in vitro models can entirely imitate the in vivo conditions, there is no perfect in vitro model of the BBB. Therefore, it is important to choose the in vitro model according to the requirement of the study. More details about the advantages and disadvantages of the different in vitro BBB models are currently covered in a thorough review article by Bagchi et al. (2019) [19]. 

### 3.2. In Vivo Methods

In contrast to in vitro methods, various in vivo methods have been employed to determine the kinetics of drug transport across the BBB. These include intravenous injection, in situ brain perfusion, microdialysis, quantitative whole-body autoradiography (QWBA), and molecular imaging such as single-photon emission computed tomography (SPECT), positron emission tomography (PET), and optical imaging. Brain perfusion is the most widely used technique for obtaining in vivo permeability values for drugs [24,25]. As such, brain perfusion allows injection of a solute into the brain vasculature at higher flow rates and solute concentrations than can be achieved by systemic circulation and hence allows a wider range of solute permeabilities to be measured at a fixed perfusate concentration. Direct injection of the solute into the brain minimizes metabolic loss and plasma protein binding. In this technique, the common carotid artery is cannulated and connected to a perfusion system. Immediately after the animal’s heart is stopped, the molecule of interest dissolved in a physiological solution is infused into the brain typically for 5–300 s. Subsequently, the brain is removed, and the ipsilateral hemisphere is dissected, weighed, and the solute concentration determined by chromatography (LC-MS, HPLC, GC) or by radioactive counting methods (gamma or liquid scintillation counting) if the drug is radiolabeled. 

In vivo microdialysis is another well-established quantitative technique in neuroscience for measuring small molecule concentrations in brain interstitial fluid (ISF) and CSF with minimal invasion into live animals. This technique essentially began with the push–pull method, which examined the possibility of using a semi-permeable membrane to sample free amino acids and other electrolytes in neuronal extracellular fluid. The technique was further improved by the development of the dialysis bag as a means of collecting the dialysate [26]. Since multiple microdialyis probes can be implanted in the brain, the disposition of drug within different regions of the brain can be simultaneously characterized. The use of this technique to measure macromolecule concentrations in brain, however, has been very limited. This is mainly due to the lack of availability until recently of large molecular weight cutoff (MWCO) probes and the need for a complicated push–pull system to perform microdialysis with large pore probes [27]. Although the push–pull microdialysis procedure for antibodies is challenging and requires extensive training, recent studies have shown that it can provide direct in vivo measurement of free antibody concentration in selected regions of the brain in freely moving animals [28,29]. This technique can avoid the detection of bound antibodies to the brain capillary endothelial cells and the neurons, and readouts of free antibody concentration in the brain interstitial ISF tend to better represent the required therapeutic concentration at the site-of-action in the brain. The theory and underlying general principles of in vivo microdialysis in general and brain microdialysis in particular are discussed in a review by Darvesh et al. [26].

On the other hand, to evaluate the in vivo PK and tissue distribution of antibodies, intravenous injection of radiolabeled antibody followed by collection of blood and tissue samples from the CNS at different time points (“cut and count”) can be used as assays for sensitive uptake analysis [30]. Such an approach, however, is tedious and requires a large number of animals to ensure the reproducibility and reliability of the results. Today, QWBA, which relies on the use of X-ray film and phosphor imaging technology or radioluminography, is another standard method for conducting tissue distribution studies throughout the body of laboratory animals. These studies suggest that QWBA helps study the spatial and regional differences in areas as fine as 50–100 μm and is a good method for studying the targeted delivery of therapeutic proteins across the BBB [31,32]. The main advantage of QWBA is the minimal sample processing at true tissue-level (as opposed to organ-level) concentrations from an in situ preparation. 

Furthermore, the continuing development of high-resolution PET and SPECT scanners and the availability of suitable radionuclides (e.g., Cu-64, Zr-89, In-111, I-131, I-124) are providing a non-invasive in vivo alternative that simplifies considerably the visualization and measurement of the whole body and organ PK, as well as brain uptake of antibodies. In this way, real-time dynamics can be obtained on whole body biodistribution of radiolabeled antibodies in the same animal or patient. The major advantages of these radionuclide-based molecular imaging techniques (SPECT and PET) are that they are very sensitive (down to the picomolar level), quantitative, and there is no tissue penetration limit. As a result, new applications of brain molecular imaging in animals are continually being established, which show a correlation between brain uptake of radiolabeled antibodies and brain target levels [33,34,35,36]. Another advantage is that molecular imaging methods have good spatial resolution (0.35–1.5 mm), allowing differentiation of tracer uptake on the suborgan level. Accordingly, the importance of spatial resolution in understanding therapeutic protein distribution within the brain has been the subject of several studies in which differences in brain penetration and distribution related to drug format are characterized [30,37,38]. 

Thus, significant effort has been made to radiolabel protein drugs with radionuclides, which in turn provides a method for tracking the location and quantifying the total radioactivity in tissues. However, the main limitation, which is shared by all these in vivo studies (e.g., “cut and count”, QWBA, and molecular imaging) that rely on the usage of a radiolabeled compound, is that these technologies provide data on total radioactivity only and not specifically of the parent compound. In other words, the concentration of radioactivity does not always equate with the identity of the original compound that was radiolabeled, and it may also include radioactivity associated with metabolites and/or degradation products. The reader is referred to a comprehensive review by Tibbitts et al. (2016) of the different radiolabeling methods and the different in vivo technologies and approaches in order to gain a better mechanistic understanding of PK and protein distribution as a way to drive forward the selection of successful drug candidates [31].

In summary, in vitro BBB model selection parameters using human derived cells are critical for predicting drug transport because the disease in question may affect the barrier properties. Although many in vivo experiments have been traditionally performed, drug permeability tests are now carried mostly by in vitro BBB models due to ethical problems, differences between species, and expensive in vivo experiments. Nevertheless, a combinatorial approach of in vitro BBB models and in vivo tests will be the key to the development of CNS therapeutics with improved PK properties and better BBB penetrability [19,39].

## 4. Approaches to Optimize BBB Internalization and Uptake of Antibodies

Research has revealed that the BBB is not only a substantial barrier for drug delivery to the CNS but also a complex, dynamic interface that adapts to the needs of the CNS and responds to physiological changes [40]. Optimization of drug delivery across the BBB could be achieved by several approaches: (a) pharmacologically, to increase the passage of drugs through the BBB by optimizing the specific biochemical properties of a compound [11]; (b) by BBB modulation, which includes transient osmotic opening of the BBB; and (c) physiologically, exploiting the various transport mechanisms present at the BBB. Many biomolecules (e.g., antibodies, recombinant fusion proteins, and nanoparticles), however, cannot get through the BBB unless the permeability of the BBB is altered using modulation of the tight junctions of the cerebral endothelial cells, which can result in some serious complications [11]. Research has shown that BBB internalization and trans-barrier transport of biomolecules can be manipulated on the basis of their physicochemical characteristics [41]. As a result, it is evident that various biomolecules with different parameters and characteristics are able to transverse biological barriers dictated by the barrier’s set of limitations and specific criteria for internalization. Hence, it is expected that at some point the BBB physiology and physicochemical characteristics of antibodies will allow for the control of the rate and extent of cellular uptake, as well as the delivery of the antibody intracellularly, which is imperative for drugs that require a specific cellular level to exert their effects at the targeted site in vivo. Designing antibodies that can overcome this BBB protection system and achieve optimal concentration at the desired therapeutic target in the brain is a specific and major challenge for scientists working in CNS discovery [42]. In recent years, some progress has been made in terms of enabling the development of pharmacokinetic and pharmacodynamic (PK/PD) relationships for antibodies as therapeutic agents as well as in understanding how these relationships are influenced by target antigens and molecular properties. 

In order to enhance antibody delivery to the brain, the following strategies for delivery optimization have been explored: (i) development of BBB-crossing bispecific antibodies, which have been engineered to incorporate one specificity against a BBB RMT receptor (Table 2) and the second specificity against a CNS therapeutic target to produce a pharmacological effect; and (ii) protein engineering efforts, which allow for the customized design of antibody constructs with physicochemical, molecular, and binding properties better optimized for successful transport across the BBB. Notably, antibody uptake is highly influenced by factors such as their size, surface charge, structure, hydrophobicity, affinity, antigen internalization, and dual targeting with bispecific antibodies [40,41,43,44,45]. The previous sections discussed the different transport mechanisms for the internalization of antibodies. Taken together, this section discusses the ideal antibody characteristics when employing transport mechanisms to achieve optimal cellular uptake (i.e., achieve desirable concentration range) at the BBB. Thus, this section focuses on examining the physicochemical and functional parameters of antibodies in regard to their relations and interactions with the physiology of the BBB and how those relations and interactions both facilitate their development as outstanding therapeutics. 

### 4.1. Modification of BBB Permeability

The BBB is the first barrier that restricts the transportation of drugs from the blood to the brain. Because of this, researchers have developed various strategies to overcome or bypass the BBB, including penetration of the BBB by temporarily enlarging the BBB pore size, which could allow molecules such as antibodies to diffuse directly into brain [9]. In essence, modulating the efficacy of the tight junctions between cerebral endothelial cells so that the paracellular route of access to the brain is accessible is an applicable approach that has been utilized to permeabilize the BBB to drugs and enhance brain uptake. For instance, Neuwelt et al. (1981) discovered that mannitol, a hypertonic solution, can be administered simultaneously with drugs to enhance their delivery to brain tumors [51]. Currently, researchers are still using this strategy to deliver drugs to the CNS. Hypertonic solutions are thought to osmotically remove water from the endothelial cells, causing the cell to shrink, which may cause cellular changes that can affect the tight junctions [11]. This method is transitory, as the barrier closes within 10–20 min following BBB disruption. Unfortunately, this method is not selective for a specific drug and may increase uptake of other blood-borne molecules, such as neurotransmitters, which could be potentially harmful. Similarly, solvents such as high dose ethanol or dimethylsulfide, alkylating agents such as etoposide, alkylglycerols, and vasoactive agents such as bradykinin and histamine, have all been used to open the BBB and facilitate the delivery of drugs to the brain [52]. Since these compounds must be of a certain concentration to open the BBB, the BBB returns to its intact status when the blood concentration of these compounds falls lower than the threshold. Therefore, the dose and administration schedule must be optimized. The opening of the BBB is again presumably nonselective; thus, the use of these agents to affect BBB permeability can be highly traumatic, and could potentially cause serious side effects, such as seizures, permanent neurological disorders, and brain edema [9,11].

To circumvent these problems, focused ultrasound (FUS) and MRI are being employed as modulators of BBB function [53]. FUS has been used to enhance the delivery of various drugs to the brain, and it has been shown that the concentration of drugs in the brain hemisphere treated with FUS was approximately 3.5 times higher than the control hemisphere [53]. Combining FUS with other targeting methods could further elevate the accumulation of drugs in the brain. As an example, combining FUS with MRI targeting could improve the brain accumulation of drugs by 16-fold [54]. Although the toxicity of FUS on the brain is considered minor, and neurotoxicity was not observed, the clinical application of this method still should be viewed cautiously [55]. An advantage of these methods is that they can be focused with some precision to a particular region of the brain, thus modulating the BBB at a preferred site in order to release the drug locally. These modifications in BBB function and integrity appear to be transient and reversible, increasing the apparent safety of this method.

### 4.2. Physiological Approach to Transport Antibodies Across the BBB

Although the BBB is intact, mechanisms described in detail in Section 2 can be used to overcome this barrier. These strategies have been explored extensively over the past several decades when designing therapeutic antibodies for neurological disorders. Many of these strategies rely on receptors and carriers that are overexpressed on the BBB (Table 2), which can mediate the transport of specific ligands and their cargo. 

Large molecules necessary for the brain’s normal function are delivered to the brain by specific receptors that are highly expressed on the endothelial cells that form the BBB. This mechanism is described in the previous section as receptor-mediated transport (RMT). Additionally, the intercapillary distance in the brain is very small (on average 40 μm), and every neuron is virtually perfused by its own blood vessel, making these receptors abundant at the BBB [33]. Antibodies can be modified to be able to passage the BBB by conjugation to ligands that recognize receptors expressed at the BBB. This strategy in fact is the most effective way of delivering antibodies through the BBB and into the brain. This physiological approach targets IR, TfR, LRP-1 and 2, and other receptors. Overall, therapeutic compounds are able to cross the BBB after association with these specific ligands, forming “molecular Trojan horses” (Table 3). Proof of concept studies have demonstrated that TfR-specific antibodies bind to the receptor on the endothelial cells and allow the associated therapeutic agent to cross the BBB via receptor-mediated transcytosis, making TfR particularly promising in brain-targeted delivery [56]. Modifications are still being made in the use of TfR as a delivery system after studies showed that antibodies bound to the TfR were retained in the brain endothelium and did not penetrate into the CNS. To address this problem, a “brain shuttle” approach has been developed that fuses the C-terminus of a monoclonal antibody against Aβ, the peptide that accumulates in the brain of AD patients, to an anti-TfR Fab, which facilitates the BBB transcytosis of an attached immunoglobulin [57]. This differs from current approaches where the TfR antibody carries a therapeutic cargo or a bispecific antibody with optimized binding to TfR that targets the enzyme β-secretase (BACE1) associated with AD [58,59]. Compared with the monospecific anti-BACE1 antibody, the bispecific antibody had increased accumulation in the brain and led to an increased reduction in Aβ levels [60].

Multiple studies have extensively documented the use of the insulin receptor (IR) for the targeted delivery of drugs to the brain using specific antibodies directed against IR [46,66]. Animal studies have shown that total brain uptake of the anti-human IR is 4% of injected dose at 3 h post injection and confirmed that it is able to transport an associated molecule across the BBB. Furthermore, applications of the TfR and IR antibodies to a molecular Trojan horse for the delivery of therapeutics have been documented where different forms of conjugated and fusion proteins have been generated [33]. LRP-1 and 2 expressed on neuronal cells have also been exploited to deliver drugs to the brain in a similar fashion as TfR and IR [67]. For now, these receptor antibodies described above may not be the only answer to the biologics brain targeting question [68]. Regardless, the substantial research performed with these available antibodies has provided invaluable insight on the mechanisms of action of receptors at the BBB and has also helped to highlight protein engineering issues that must be addressed (as presented below) in order to develop a successful approach for transporting therapeutic antibodies across the BBB.

### 4.3. Antibody Engineering Approaches to Increase Trans-BBB Transport 

A serious limitation to the use of many antibodies in the design of improved biotherapeutics is their non-optimal behavior in the organism, including their poor PK parameters, non-optimal distribution, inhibition of binding with FcRn, and toxicity [69]. At the same time, one of the main problems is their frequent administration at a large dosage, which increases the risk of immunogenicity and side effects and reduces patient tolerance for the antibody. As such, one should note that antibody production is a continual design process that involves the generation and optimization of antibodies to enhance their clinical potential [70]. Moreover, much of the development and clinical experience that is gained from the generation and optimization of one antibody is applicable to other antibodies, thereby streamlining certain activities and decreasing some of the risks that are intrinsic to drug development. For example, to optimize the properties of an antibody for a particular indication, it would be preferable to improve or even delete particular characteristics. As an example, one major goal in developing therapeutic antibodies in neurological diseases is to improve the clinical utility of these reagents with respect to antigen targeting and better brain uptake (i.e., better brain-to-blood ratio) to encourage effective disease therapy. Achieving this goal depends not only upon a thorough understanding of molecular properties underlying antibody behavior and function but also upon the development of techniques to manipulate these properties in such a way that enhances their therapeutic potential [43]. For instance, PD response is often directly proportional to brain exposure and, thus, plasma half-life. As such, a typical goal in biotherapeutic development is to identify a candidate molecule having desirable PK properties or, alternatively, to manipulate a molecule’s properties to improve its PK while preserving antigen recognition. PKPD properties of antibodies are governed by both molecule-dependent and species-dependent parameters. Biological processes such as antigen binding and receptor binding are important determinants in antibody PK. Since the field of therapeutic monoclonal antibodies has become extremely competitive, especially against validated antigens, it is necessary to develop highly optimized antibodies, above and beyond humanization [70]. A highly optimized humanized antibody would have superior pharmacological properties important for clinical efficacy, such as high antigen-binding activity and long half-life, as well as biophysical properties important for commercial development of the therapeutic antibody, including stability and expression yield in host cells. In order to generate such highly optimized antibodies, it is necessary to consider these pharmacological and biophysical properties during the process of humanization and manufacture. This section describes the critical properties of therapeutic antibodies that should be sufficiently qualified, including Fc and antigen binding affinity, and physiochemical properties and PK.

Fortunately, antibody fragments, such as single-chain Fv, diabody, triabody, Fab, F(ab′)2, and full length antibodies, ranging in size from 30 to 150 kDa and valence from one to three binding sites can be also derived via molecular engineering [71]. The single chain Fv (scFv, 30 kDa) is one of smallest forms of antibody that consists of variable light and heavy domains connected by a flexible peptide linker of approximately 15 amino acids generating one binding site. Diabodies (60 kDa) consist of two single chains joined by a very short linker, while triabodies (90 kDa) do not have a linker, thereby forcing trimerization. For example, the ability of these fragments to bind to tumor lies in a fine balance between their ability to penetrate tumor tissues due to their small size and their fast clearance from the body by the kidneys [72]. While retaining their antigen-binding capabilities, these fragments not only cleared faster but were also shown to have much higher tumor/organ ratios compared with their larger counterparts [72]. These antibody fragments are also used in neuroimaging agents in various diseases [35,73]. In this aspect, these studies showed that a diabody and a triabody penetrate the brain parenchyma more rapidly than the full length antibody, which in turn enables in vivo imaging of Aβ pathology at an earlier time point after administration [73].

#### 4.3.1. Role of Fc Receptors

Receptors on the blood–brain barrier bind ligands to facilitate their transport to the CNS. Therefore, it is hypothesized that by targeting these receptors, therapeutic macromolecules (e.g., nanoparticles, antibodies) can be delivered to the CNS [74]. In this regard, FcRn, LRP, TfR, and IR receptors play an important role in regulating the endocytosis and transcytosis of IgGs, peptides, and proteins across the BBB (Table 2) [12]. The function and mechanism of FcRn in regulating IgG recycling have been well characterized. Because of the protective effects of FcRn against lysosomal degradation of IgG, generating Fc fusion proteins and modulating the pH dependent affinity between Fc and FcRn has been utilized to improve the PK of therapeutic antibodies [74]. In vitro studies have shown that FcRn regulates the transport of IgGs in both directions across the endothelial barriers of blood vessels, including those in brain, intestine, and placenta [75]. Importantly, studies using immortalized rat brain endothelial cells suggested that the human Fc fragment transports faster in the brain-to-blood direction than in the opposite direction [76]. These studies showed that while FcRn mediated the transport of IgGs across peripheral vascular cells in both directions, FcRn only mediated transport across BBB in the brain-to-blood direction. Modulating the interaction between Fc and FcRn through protein engineering has been applied to improve the PK of the therapeutic antibodies. Various studies have shown that the prolonged half-life and exposures of therapeutic antibodies can be achieved by increasing the pH-dependent binding affinity between Fc and FcRn [74]. 

There have been controversial studies on the role of FcRn in regulating the efflux of IgG from the brain and whether FcRn behaves as an efflux receptor that can transport antibodies across the blood–brain barrier back into the systemic circulation. One study in rats suggested that BBB FcRn mediates the efflux of IgG from the brain to the blood [77]. This study showed this efflux mechanism can be avoided when using antibody fragments devoid of Fc regions (Fab, F(ab’)_2_ and scFv fragments). Subsequently, another study investigated the mechanism of Aβ immunotherapy in the clearance of Aβ amyloid peptide in APPsw mice, a model that develops Alzheimer’s disease-like amyloid pathology [76]. The study showed that anti-Aβ IgG-assisted efflux of Aβ amyloid peptide from the brain to the blood in wild-type mice was inhibited when the FcRn gene was knocked out. Taken together, these data suggest that FcRn at the BBB may play a role in regulating IgG-assisted Aβ amyloid peptide removal from the aging brain. 

Unfortunately, other studies have shown that brain distribution and disposition of IgG is not regulated by FcRn and FcγR [78,79]. In these studies, IgG was injected intravenously to FcRn knockout and control mice [78]. As anticipated, the plasma clearance of IgG was increased by about 10 times, and the plasma exposures decreased by 4–5 times in FcRn deficient mice when compared with the controls. The brain exposure of IgG, however, was also reduced to a similar extent, and as a result, the brain-to-plasma ratios of IgG were not significantly different between the FcRn deficient and the controls. In another study, the role of FcRn in regulating brain IgG disposition was further investigated in the FcRn knockout, FcγR knockout, and control mice [79]. Compared with controls, the plasma and brain exposures from FcγR knockout mice were not significantly different, and the plasma and brain exposures from FcRn knockout mice decreased by 3–4 times as anticipated. However, similar to what was observed in the previous study, the brain-to-blood exposure ratio was not significantly different among the knockout and control mice. Together, these two studies indicate that FcRn and FcγR do not contribute to the “BBB” that limits IgG uptake into the brain. Similar to what was reported by Garg et al. and Abuqayyas and Balthasar, recent results from other groups showed that there was no IgG uptake difference between FcRn knockout and wild-type mice in the brain, which suggested that FcRn has little effect on the distribution of IgG in the brain [80]. In support of these findings, a more current study evaluating IgG uptake in tissues for FcRn wild type and FcRn^-^ constructs indicates that FcRn does not contribute significantly to the brain for IgG in mice [81]. This study also demonstrates that FcRn does not play a protective role in the brain. These data are not consistent with previous studies that showed higher brain uptake for the engineered high-IgG FcRn binder relative to the wild type [82]. As postulated above, higher brain concentrations of the IgG variant with enhanced binding to FcRn could result from a role in efflux of IgG, as opposed to influx [76,77]. Further evidence toward this notion is the fact that the brain expression of FcRn is co-localized with the glucose transporter 1 (Glut1) in the capillary endothelium, suggesting that FcRn is expressed in the proper location to potentially mediate reverse transcytosis of IgG from the brain to the blood [83]. In summary, there is no consensus on the role of FcRn in influencing the blood-to-brain transcytosis of IgG across the brain endothelial cells (BECs) despite several notable studies. Indirect evidence of potential FcRn-mediated recycling was provided by recent studies demonstrating that IgG transcytosis across an in vitro BBB exhibits a non-saturable and nonspecific mechanism and supports the use of RMT approaches or modifications of biophysical properties, such as pI, to achieve improved brain uptake of therapeutic IgGs [7,84].

#### 4.3.2. Role of Antigen Binding

Trans-BBB delivery methods that use targeting antibodies are often hampered by limited flux through the BBB. A solution to this problem lies in the rational engineering of BBB-targeting antibodies. Leveraging knowledge of intracellular trafficking, researchers have begun to tune selected binding properties of the antibody–antigen receptor interaction. Engineered binding affinity, avidity, and pH sensitivity have been shown to affect binding, intracellular sorting, and release, ultimately leading to increased brain uptake of the targeting antibody and its associated cargo [7]. The first successful attempts for chimeric proteins targeting cell receptors initially relied on cationized albumin, which lacked brain selectivity, and then later IgGs directed against IR or TfR receptors [85]. However, the success of these initial antibodies was limited by their high affinity, which hindered an efficient release and penetration into the brain parenchyma [58]. A variety of protein shuttles have been investigated; most of them are ligands of receptors on the brain endothelium that compete with their endogeneous proteins (e.g., apolipoproteins A and E, receptor-associated protein, transferrin, lactotransferrin, melanotransferrin, and leptin). Although a few non-endogenous proteins have been used (e.g., wheat germ agglutinin, non-toxic mutant of diphtheria toxin), they also have shown moderate efficacy and selectivity [13]. Recent efforts have leveraged antibody engineering strategies to increase trans-BBB transport and have highlighted the importance of the antigen-binding and trafficking issues. As shown in Table 1 and Table 2, each targeted receptor may exhibit differential responses to engineered binding properties, illustrating the need to better understand antibody–antigen receptor interactions and trafficking dynamics.

**Figure 1:**
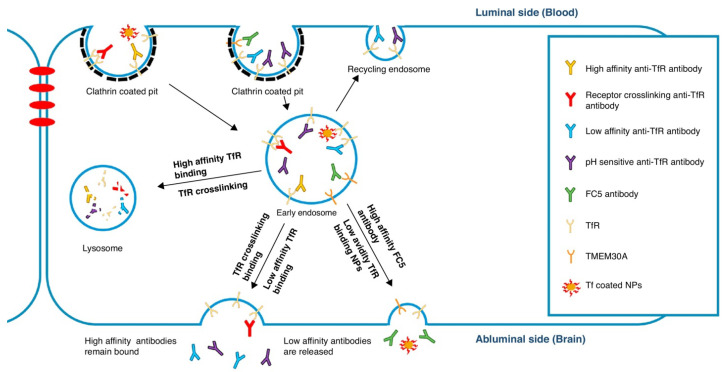
A schematic depiction of the various engineering optimization strategies for increased transcytosis of antibodies and nanoparticles (NPs) across the BBB. High-affinity monovalent and bivalent anti-TfR antibodies internalize readily into the early endosome (EE) but then direct the antibody–receptor complex toward lysosomal degradation, possibly by crosslinking the TfR and altering its intracellular trafficking. While high-affinity monovalent anti-TfR antibodies can transcytose the BBB, they remain bound to the receptor on the abluminal side, limiting the dose to the brain. In contrast, low-affinity anti-TfR antibodies decrease antibody-TfR sorting to the lysosome and can either be recycled back to the luminal side or are transcytosed to the abluminal side where they dissociate from TfR, leading to increased brain accumulation. Similarly, Tf-coated nanoparticles show a higher transcytosis capability when lowering the Tf coating content, resulting in reduced avidity. Further, pH-sensitive TfR-binding antibodies that can dissociate from TfR in the acidic EE lead to increased transcytosis compared with pH-insensitive antibodies. In the case of the single domain antibody FC5, increased affinity toward the receptor leads to an increase in the amount of transcytosed antibody, highlighting the fact that vectors utilizing different trafficking machinery may require customized optimization. This figure is reproduced from Goulatis and Shusta (2017) with permission of the copyright owner [7].

**Figure 2:**
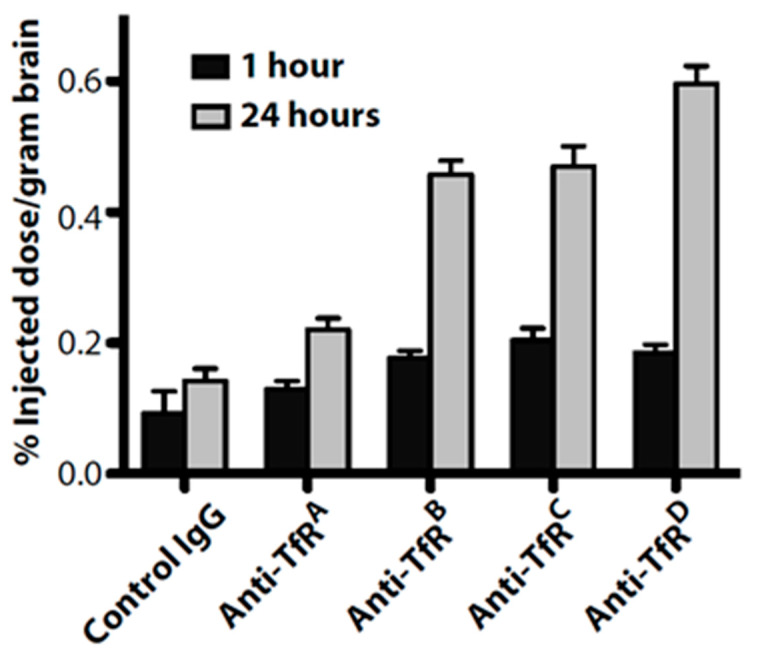
Lower-affinity anti-TfR^D^ antibodies (A > D) at therapeutic doses show increased brain uptake. This figure is reproduced from Yu et al. (2013) with permission of the copyright owner [58].

Regarding the above, well-designed experiments have engineered the binding properties of anti-transferrin (anti-TfR) antibodies to study their trafficking and delivery in vitro and in vivo. First, antibody affinity and avidity for TfR were evaluated, and it was shown that higher brain uptake of anti-TfR antibodies can be accomplished by lowering antibody affinity [58]. Intravenous administration of antibodies having a range of affinity to TfR (Kd = 6.9–111 nM) indicated that at trace doses, mouse brain uptake directly correlated with affinity, suggesting that receptor engagement at the blood side of the BBB was the key parameter governing uptake (Figure 1). This figure shows the diagram taken from Goulatis and Shusta (2017) to illustrate that high-affinity monovalent and bivalent anti-TfR antibodies internalize readily into the early endosomes but then direct the antibody–receptor complex toward lysosomal degradation, possibly by crosslinking the TfR and altering its intracellular trafficking [7]. While high-affinity monovalent anti-TfR antibodies can transcytose the BBB, they remain bound to the receptor on the abluminal side, limiting the dose to the brain. In contrast, low-affinity anti-TfR antibodies decrease antibody-TfR sorting to the lysosome and can either be recycled back to the luminal side or get transcytosed to the abluminal side where they dissociate from TfR, leading to increased brain accumulation. Further, pH-sensitive TfR-binding antibodies that can dissociate from TfR in the acidic endosome led to increased transcytosis compared with pH-insensitive antibodies. In the case of the single-domain antibody FC5 (Table 3), increased affinity toward the receptor leads to an increase in the amount of transcytosed antibody, highlighting the fact that antibodies utilizing different trafficking machinery may require customized optimization (Figure 1). However, at therapeutic dosing (20 mg/kg), an inverse correlation was observed, where the lowered affinity antibody demonstrated greater brain accumulation (up to 0.6% ID/ g) (Figure 2). These data demonstrate that lowered affinity allows for antibody release from the TfR at the abluminal membrane, while higher-affinity variants remain bound to the TfR. Further studies provided the evidence that affinity-derived effects on brain uptake of anti-TfR antibodies were at least in part caused by altered intracellular trafficking and lysosomal degradation [37]. The high-affinity anti-TfR antibody was found to be more prominently trafficked to the lysosome and degraded, resulting in reduced cortical TfR levels. Thus, productive trans-BBB anti-TfR antibody trafficking could be increased by lowering antibody affinity. 

In addition to studies of TfR antibody-binding affinity, the role of avidity has also been explored with similar conclusions. In order to investigate avidity effects on trans- BBB transport, a Fab fragment targeting the TfR was fused to the carboxy-terminus of an anti-BACE1 (β-amyloid cleaving enzyme-1) antibody in a bivalent (dFab) or monovalent (sFab) format [57]. Monovalent binding of an anti-TfR antibody allowed for preferential transcellular transport in brain endothelia, while bivalent binding led to diversion of trafficking toward the lysosome. The results of this study strongly suggest that differences in TfR-binding mode led to major differences in intracellular trafficking, which ultimately allows sFab-associated cargos to cross the BBB. Similarly, another in vitro study has investigated whether or not pH-insensitive TfR binding could be an additional engineering approach for regulating anti-TfR antibody trafficking and increasing trans-BBB transport [86]. These data demonstrated that attenuated binding of an anti-TfR antibody at endosomal pH can lead to differential intracellular trafficking, ultimately enhancing transcytosis across the BBB.

#### 4.3.3. Role of Biophysical Properties

The majority of small drugs that are used to treat CNS disease have a molecular weight between 150 and 500 Da [11]. This does not indicate that drugs with a molecular weight less than 150 or greater than 500 Da are unable to cross. Characteristics that reduce the ability of small molecules to cross the BBB include a polar surface area in excess of 80 A ° and a high potential for hydrogen bond formation. Additionally, increased number of positive charges and increased flexibility contribute to BBB crossing. Lipid solubility is a clear indicator of small drugs that can pass through the BBB [87]. Rules for proteins have some similarities and some apparent differences from those for small drugs [11]. Most proteins are poorly soluble in lipids and so would not be expected to penetrate the BBB very well by trans-endothelial diffusion. However, lipid solubility was a predictor of BBB penetration for one series of peptides and proteins that had molecular weights ranging from 486 to 6000 Da. The largest substance found to cross the BBB using transmembrane diffusion thus far is cytokine-induced neutrophilchemoattractant-1, which has a molecular weight of 7800 Da [88]. This is thought to represent a direct correlation between BBB penetration and the ability of a drug to deliver cargoes across the cell membrane. One of the primary factors in determining whether a protein will cross the BBB is its lipophilicity. A strategy for enhancing the ability of a peptide to cross the BBB is increasing its lipophilicity. There are a number of techniques able to do this, including alteration of the protein structure, methylation, halogenation, or acylation. Structural changes, for example covalently binding the drug to lipidic moieties, such as long chain fatty acids, will increase the lipophilicity of a peptide [89]. Peptides with a high number of hydroxyl groups tend to promote hydrogen bonding with water, which leads to a decrease in membrane permeability. Decreasing hydrogen bonding therefore increases membrane permeability. Ideally, there should be potential for the formation of fewer than eight hydrogen bonds when developing new drugs. Methylation is one method used to reduce hydrogen bonding. This illustrates an important point for protein modifications: that the location and type of modification play a significant role in improving BBB transport of your peptide of interest [11]. Halogenation of peptides and proteins can also lead to increased lipophilicity and BBB permeability. The increase in BBB transport of peptides was dependent on which halogen was utilized; chloro and bromo additions increased BBB transport, while fluoro and iodo additions had no effect [90]. An alternative approach is acylation of the N-terminal amino acid, which can increase the lipophilicity of peptides and proteins. For example, acylation of insulin improved its ability to cross the BBB while maintaining its pharmacological effects. Another approach involves glycosylation and hyperglycosylation of therapeutic proteins [91]. In this case, the in vivo results showed that glycosylation is required to maintain protein exposure in blood and proved to increase protein uptake into the CNS [92].

Along these lines, the large change in biophysical properties induced by therapeutic cargoes and the distinct location of the targets of these drugs inside the brain has limited the universal aspiration of most BBB shuttles [13]. Hence, in general, each protein shuttle is prominent in the delivery of a particular family of cargoes. There are many receptors and carriers that are overexpressed on the BBB (Table 2), which can mediate the transport of specific ligands and their cargoes. Additionally, the membrane of the BBB is negatively-charged and shows high affinity with positively charged compounds, which could also trigger the internalization by cells [9]. Thus, these kinds of ligands could mediate the penetration of macromolecules through the BBB. Efficient transport of macromolecules across the BBB through endocytic mechanisms involves both specific (receptor-mediated transcytosis) and/or nonspecific (adsorptive-mediated transcytosis) interactions with proteins and receptors expressed on the brain endothelial cell surfaces. In adsorptive-mediated transcytosis, endocytosis is generally promoted by the interaction of the often positively charged molecule with membrane phospholipids and the glycocalyx [13]. The most common approach relies on enhancing positive charge, in order to mediate interaction with the anionic glycocalyx. However, this approach leads to higher unspecific uptake in many other tissues, often resulting in off-target effects, which in addition requires a high degree of tailoring for certain small molecules that are rarely applicable to biotherapeutics. Another approach for drug delivery to the CNS, as shown in the previous section, focuses on BBB shuttles that allow the transport of a wide range of molecules, comprising small molecules, proteins, nanoparticles, and IgGs across the BBB. Substrates of natural carriers such as glucose and neutral amino acids have been applied to transport small molecules through their natural carriers on the BBB, while for biomolecules the focus has been set on receptor ligand proteins (Table 2) since endocytic pathways tolerate a high cargo load [13].

A common goal for therapeutic antibodies is to extend plasma half-life as a means to increase exposure, often expressed in terms of the area under the plasma concentration-time curve [74]. In contrast, cationization tends to shorten plasma half-life due to an enhancement in both the rate and the magnitude of tissue distribution. However, cationization might prove to be a useful strategy in specific applications in which prolonged antibody exposure may be sacrificed for the sake of rapid, enhanced tissue uptake (e.g., targeting antibodies to efficiently cross the BBB) [93]. Cationization of antibodies has also been explored as a means to encourage extravasation, antigen binding, and receptor-mediated endocytosis of antibodies into target cells [94]. In contrast to native Abs, which are generally excluded from cell membranes in the absence of receptor-mediated endocytosis, cationized Abs are better able to reach the intracellular space via absorptive-mediated transcytosis (AMT). Electrostatic interactions between positively charged proteins and negatively charged cell membranes could permit cell entry via nonspecific membrane flow and have been implicated in the mechanism by which cationized antibodies are rapidly endocytosed by cells in vitro. A similar phenomenon is suggested to induce absorptive-mediated transcytosis across microvascular endothelial barriers in vivo [94]. This interaction also occurs further with sialic acid moieties on the luminal surface and heparin sulfate group on the abluminal surface. AMT of cationized albumin is triggered by this electrostatic interaction and results in the transport of the moiety across the BBB [93]. The use of cationized albumin for the transport of β-endorphin, a very large molecule that cannot cross the BBB, has been reported in rats [95]. After conjugation with cationized albumin brain uptake of β-endorphin was increased. When the isoelectric point of antibodies is raised from neutral to highly alkaline, cationized antibodies are formed. These antibodies are used mainly as neuroimaging agents in various diseases, including brain tumors, AD, and stroke [96]. Mechanisms governing the passage and partitioning of small molecule drugs and antibodies across the BBB have also been the subject of several reviews and modeling studies [29,97,98]. While the distribution of small molecules is influenced by multiple factors, including drug liposolubility, free vs bound concentrations in blood and brain fluids, and their (bidirectional) transport via BBB carriers and efflux pumps, some of these processes may not be important in the case of some biologic molecules [61]. For example, antibodies (including VHHs) are not substrates of efflux pumps and their “bound” concentration in body fluids can be considered negligible in most cases. Their paracellular “filtration” across the BBB is essentially completely restricted by tight junctions of brain endothelium and choroid plexus epithelium, respectively. In the absence of specific transport mechanisms such as the RMT, they therefore can access the brain only via a low-rate nonspecific adsorptive endocytosis. To discover new antigen−ligand systems for transvascular brain delivery, a recent study developed a method for functional selection of brain microvascular endothelial cell-specific internalizing and transmigrating antibodies [62] from a phage-display llama single-domain antibody (sdAb) library (Table 3). sdAbs are the VHH fragments of the heavy-chain IgGs, which occur naturally in camelid species and lack light chain, and are half the size (15 kDa) of a single-chain antibody (scFv) [99]. These sdAbs have been shown to internalize into the brain’s endothelial cell and transmigrate in an in vitro BBB model via a saturable, energy-dependent and charge-independent process; pretreatment of cells with highly cationic protamine sulfate did not affect sdAb transcytosis [100]. Similarly, two positively charged control antibodies, showed minimal “passive” transcytosis in an in vitro BBB model. As such, it can be assumed that CSF levels of control sdAb, which does not bind any known receptor in mammals, are representative of nonspecific passive uptake processes (macropinocytosis; adsorptive endocytosis) of large, hydrophilic, and positively charged biologic molecules at the BBB. 

Nevertheless, as discussed above, other antibodies and single-chain antibody fragments have also been developed as “Trojan horse” bispecific antibodies for delivery of therapeutics via RMT, including IR and TfR receptor antibodies; the extensive literature on these antibodies reports a range of their serum/brain partitions (from 0.1% to 4% ID/g vs 0.06% ID/g for IgG) [58]. Due to a lack of comparative studies using the same experimental and analytical methods as well as vast differences in size and pharmacokinetics, the direct comparison between known antibody “Trojan horses” with unmodified single-domain antibodies (sdAbs) remains difficult. These sdAbs will require further engineering for improvement of their plasma half-life and potentially their binding properties before their comparative assessment with similar antibody RMT technologies can be properly performed [61].

## 5. Conclusions and Outlook 

This review focuses mainly on the historic trends and current practices of research and development activities involving the BBB as a complex interface between the blood and the CNS, essentially for the targeted delivery of antibodies to treat neurodegenerative diseases. It is well established that nearly 0.1% of circulating biotherapeutics, i.e., recombinant proteins or gene-based medicines, cross the BBB [42,45]. Hence, improving brain exposure for at least some of these molecules is the ultimate goal of the brain delivery systems. It has been proposed that CNS diseases can be initiated by several mechanisms, including decreased cerebral blood flow, perturbation of transporters, BBB disruption, deformations of capillaries, and secretion of neurotoxic substances by the BBB. Thus, the BBB may have a fundamental role in brain diseases [40,98]. Nonetheless, the BBB is intimately involved in crosstalk with the rest of the CNS and peripheral tissues and is crucial for normal brain pressure and functioning, and therefore, perturbation of its function might have physiologic consequences. As shown in Table 2 and Table 3, different physiological approaches are used to deliver biotherapeutics in the brain parenchyma. Generally, the techniques used involve direct injection or infusion of therapeutic compounds into the brain or the cerebro-ventricles or the CSF. All these approaches, however, are severely limited by poor distribution into brain parenchyma [14]. In fact, the most promising new technology uses a physiological approach to take advantage of endogenous receptors highly expressed at the BBB (e.g., TfR and IR). Fundamentally, this latter approach has been employed by cells of the BBB to enhance the delivery of antibodies across the BBB by receptor-mediated transcytosis. While the physiological approach has the potential to achieve improved brain delivery and to play a significant role in the treatment of CNS disease, in vivo preclinical studies quantifying antibody levels systematically and determining antibody activity relationship in the brain is difficult [56,101]. Despite these challenges, however, current efforts are focused on developing newer generations of antibody therapeutics that can cross or otherwise interact with the BBB for optimal in vivo benefit. For now, exploiting TfR receptors for delivering antibodies across the BBB may not be the answer to the brain targeting question [68]. However, the research performed with the available anti-TfR antibodies has provided invaluable insight into the mechanisms of action of receptors at the BBB and has also helped to highlight protein engineering issues that must be addressed in order for a successful BBB shuttle to be developed. Additionally, perhaps the most challenging aspect of moving anti-TfR antibodies to the clinic is the lack of species cross reactivity observed in the available antibodies. To solve this problem, antibodies are being engineered for use in each species under investigation, or transgenic mice are generated to express human antigens that tolerize them to humanized antibodies, something that will add significantly to development costs. 

**Figure 3:**
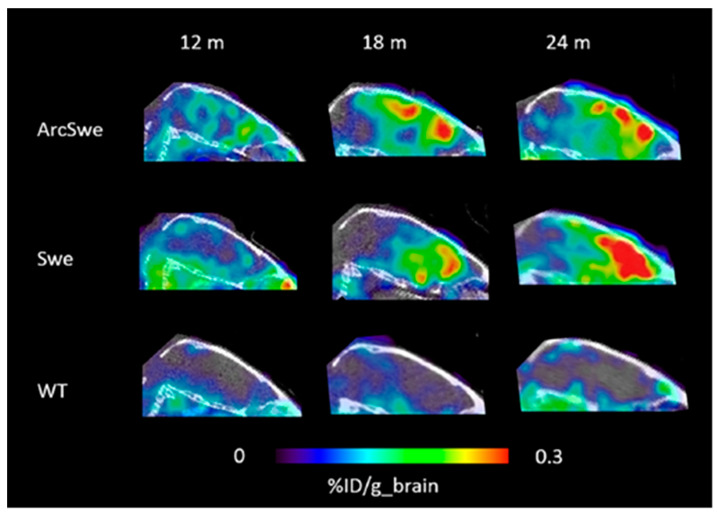
Sagittal PET images obtained at 3 days after administration of the bispecific antibody radiolabeled with I-124 in two transgenic mouse models of AD (ArcSwe and Swe) and wild-type (WT) mice at different ages (12, 18, and 24 months). Quantification of the radiolabeled antibody in brain tissue showed an increasing signal intensity with age (i.e., with increasing Aβ pathology) in the two transgenic AD animal models, while brains of WT mice were devoid of signal regardless of age. This figure is reproduced from Sehlin and Syvänen (2019) with permission of the copyright owner [73].

Moreover, in search of a solution to increase the penetration of antibodies in the brain, it is likely that all of the parameters described earlier, such as target interaction, FcRn binding, molecular size, and surface charge, are likely to play a part in the trafficking of anti-receptor antibodies across the BBB. As mentioned above, there is no consensus on the role of FcRn in influencing the blood-to-brain transcytosis of IgG across the brain endothelial cells (BECs), despite several notable studies that support the modifications of biophysical properties, such as pI, to achieve improved brain uptake of therapeutic IgGs [7,84]. Special attention is also being given to lipophilicity and overall surface hydrophobicity, since there is a tendency for those parameters to play a significant role in the BBB transport of proteins [90,92,102]. Only through the systematic evaluation of all of these parameters will it become clear which, if any, is the most important to have an effect on the PK of the brain interstitial space [103]. In the past decade, innumerable preclinical studies have been reported on the use of real-time imaging with targeted drug delivery, and this strategy has now matured with promises to assess the distribution and uptake of protein drugs [104]. For example, it has been shown that molecular imaging technologies like PET and SPECT have made important contributions to enable brain imaging of recombinant antibodies that are engineered for BBB transport, particularly in determining drug pharmacokinetics of directly labeled antibodies [35,36,105]. Additional evidence for the sensitivity of antibody PET imaging is provided by a study where a recombinant bispecific antibody was radiolabeled with I-124 and then administered in two transgenic AD and wild-type mice at different ages [73]. This study demonstrates that antibody-based PET is able to visualize and quantify early formed Aβ assemblies (Figure 3) and may become a valuable tool for disease staging of AD patients and for monitoring the effects of Aβ-directed treatment. Additionally valuable in the setting of advanced CNS imaging is the assessment of brain uptake and changes in BBB integrity, which will help to accelerate drug development by assisting in understanding and defining the challenges to translating molecularly targeted agents to the brain [10]. In this aspect, the current trend in drug discovery is to consider classical absorption, distribution, metabolism, and excretion (ADME) studies in parallel with imaging studies, enabling differentiation between biophysical and binding properties and their delivery to the brain. Such classical rules have the advantage of being very simple, as well as being easy to interpret. Their drawback, however, is that they do not take into consideration uncertainties in measurements and calculations as well as the pharmacological effect and toxicity requirements. Meanwhile, the release of an antibody drug in the brain should be accurately monitored and controlled in situ or in real-time [106,107]. It is also important to keep in mind that although no single technology currently provides all the answers, integrating different modalities into other in vivo methodologies (e.g., QWBA, microautoradiography, PET, and SPECT) can enhance our quantitative understanding of spatial brain distribution [32,36]. Furthermore, the brain is a highly vascularized organ with a relatively high proportion of endothelial cells. In determining the concentration of a therapeutic protein in the brain parenchyma, it is critical to ensure that the methods used for assessing distribution are capable of distinguishing endothelial uptake from parenchymal uptake [31]. Thus, this approach indicates that it would be more appropriate to establish a high-quality preclinical assessment of ADME, PK, and safety/toxicity studies with an emphasis on activity in the CNS, which will permit the parallel optimization of pharmacological response and druggability properties.

In addition to classical ADME studies, generation of in silico physiologically based pharmacokinetic (PBPK) models by incorporating PKPD data and safety profiles as a tool for the treatment of CNS diseases has attracted great interest from pharmaceutical scientists and are likely to be crucial to the development of novel antibody-based therapeutics [28,29,108,109,110,111]. Further, the high-throughput and low-cost nature of these models permit a more streamlined drug development process in which the identification of antibody structural optimization can be guided based on a parallel investigation of CNS uptake and safety, along with activity [112]. Hence, the development of in vivo and especially in silico models can be an instrumental tool for predicting the association between BBB penetration and the profile of expected human response for a specific antibody drug against a specific target. This approach will greatly help to simplify the practical difficulties and circumvent potential ethical controversies [106]. In essence, by simultaneously optimizing the antibody molecule in the light of their biophysical and molecular properties, BBB penetration, and activity, it should prove possible to identify a highly qualified clinical candidate and consequently enable faster development of therapeutic antibodies [70]. Although delivery of antibodies to the CNS shows great promise, a greater understanding of CNS physiology and pathophysiology is still needed. Accordingly, it would be vital to characterize further the physiological and vascular attributes such as perfusion, blood volume, and permeability to protein drugs in various CNS compartments [30,43]. Nevertheless, it is believed that the various methods historically used to assess BBB permeability or dysfunction in mouse models of human disease have led to many disparate findings. For example, early studies showed that the BBB is disrupted in Alzheimer’s disease models, potentially increasing drug permeability [113]. However, more recent data have shown that the BBB remains intact in multiple preclinical models of Alzheimer’s disease (AD) [38]. Thus, the current lack of in vivo validation of BBB permeability in neurodegenerative diseases and the lack of controlled studies hinder the understanding of antibody delivery through the BBB. Moreover, robust characterization of preclinical disease models is necessary for predicting drug delivery to target tissues and for interpreting correctly the pharmacodynamic responses to disease-modifying biotherapeutic candidates. 

After a decade of intensive engineering of antibodies followed by preclinical testing, scientists are still seeking a variety of strategies for optimizing their use as powerful therapeutic agents, particularly for targeted delivery to the CNS for the treatment of neurological disorders. With recent advances in scaffold design, construction, and selection methodologies, there is now a rapid process for recombinant synthesis of specific antibodies differing in affinity and molecular properties against virtually any BBB target [49,114]. For example, in vivo studies showed an enhanced brain uptake of antibodies with novel BBB targets but doing so, while remaining in the range of manageable safety and efficacy, is still challenging [115]. In the case of antibody modifications, it remains to be seen whether modern genetic engineering may affect the pharmacokinetics, biodistribution, therapeutic index, or safety profiles due to immunogenicity [116]. As a consequence of all these minor modifications, immunogenicity concerns are still under investigation by drug regulatory agencies, since their potential for immunogenicity can alter ADME properties, thereby greatly confounding the interpretation of PK/PD assessments [116,117].

To date, great progress has been made with the brain shuttle approach, which has proved to be successful in improving the CNS exposure of some of the large molecules with poor brain permeability, such as bispecific TfR antibodies [4,108,109]. However, further developments are still needed for this approach to become a more robust technology. Until then, fine-tuning of the biophysical and binding properties for optimal brain exposure will remain a staple of CNS drug discovery and development [42]. More importantly, in order to increase further our knowledge regarding the effects of antibody modification on brain-targeted uptake and efficacy, additional clinical studies using relevant animal species and disease models need to be implemented [118,119]. Finally, MRI-guided FUS delivery to the CNS still has great promise and provides the opportunity to improve biotherapeutics’ bioavailability locally and to improve their therapeutic profiles. In summary, targeted CNS biotherapeutics is an ever expanding and challenging but important field of study [120,121]. In fact, until now, there is only one human monoclonal antibody aducanumab (under the brand name Adulhelm^TM^), that has been FDA-approved for the treatment of people with AD. The approval was granted this year based on data from clinical trials demonstrating that aducanumab targets aggregated forms of β-amyloid, a biomarker that is reasonably likely to predict clinical benefits. Thus, since more investigators across academia and industry have joined the race to increase the uptake of antibodies across the BBB, there is good reason for optimism for additional FDA-approved CNS biotherapeutics in the near future.

## Figures and Tables

**Table 1:** Overview of large biomolecules in current preclinical development for enhanced delivery across the BBB. Part of this table is reproduced from Tucker (2011) with permission of the copyright owner [1].

Key Classes and Functions of Biomolecules
Single-domain brain-targeting antibody fragments derived from llama antibodies; led to discovery of TMEM30A, a selective BBB receptor [6,7]
2. RMT delivery of decoy receptor antibodies facilitated by fusion with an antibody to any BBB receptor leading to an elevation of drug concentration in the brain [8]
3. Bidirectional vectors, comprising one part for entry into brain by RMT and a second part to exit the brain via a second receptor-mediated BBB transport system [8]
4. Fusion antibodies for bi-directional transport across the BBB [8]
5. Delivery of a drug to the brain via a drug-loaded liposome decorated with appropriate vectors [7]
6. Synthetic low-density lipoprotein (LDL) containing cloned apolipoprotein (Apo E), for delivery of a drug across the BBB [9]
7. Liposome and poly(lactic-co-glycolic acid) (PLGA) nanoparticles coated with specified surfactants and loaded with drug for delivery across the BBB [9]
8. Nanoparticles with covalently coupled Apo E for delivery across the BBB [9]
9. A combination product comprising drug and apolipoprotein for delivery of drug to the brain and where the drug and lipoprotein can be delivered simultaneously, separately, or sequentially by intravenous injection [10]
10. Conjugates of drug with specified polypeptides derived from aprotinin, designed to increase the potency or modify the pharmacokinetics of the drug [11]
11. Conjugates of nucleic molecules and specified polypeptides from aprotinin for delivery across the BBB [11,12]
12. Specified peptides from the rabies virus glycoprotein (RVG) linked to a carrier that contains the drug for delivery across the BBB [6,13]
13. A conjugate comprising an antiviral agent with a CRM197 ligand for a receptor [7,14]

**Table 2:** Receptor-mediated targets (RMT) for transport at the blood–brain barrier. Part of this table is reproduced from Gao (2016) with permission of the copyright owner [9].

Receptors
Transferrin receptor (TfR) [12]
2. Insulin receptor (IR) [46,47]
3. Low-density lipoprotein receptor–related protein (LRP) [12]
4. Nicotinic acetylcholine receptor [9]
5. Insulin-like growth factor receptor [9,48]
6. Diphtheria toxin receptor [7,14]
7. Scavenger receptor call B type [48]
8. Leptin receptor [13,49]
9. Neonatal Fc receptor (FcRn) [50]

**Table 3:** Selected new peptides and antibodies with specific ability to cross the blood–brain barrier.

Biomolecules
Angiopep-2, a peptide ligand of low-density lipoprotein receptor-related protein 1 (LRP1), with high permeability across the BBB [12,61,62]
2. Angiopep-2-conjugated systems by conjugating the therapeutic peptides and proteins to Angiopep-2 for efficient brain delivery [12,63]
3. Two single-domain antibodies (sdAb), FC5 and FC44, were cloned using a phage-display library of llama single-domain antibodies. Owing to specific and high permeability across the BBB, FC5 and FC44 could be developed as the vectors for brain delivery [12,64,65].
4. Molecular Trojan horse: fusion of the therapeutic proteins to the monoclonal antibodies against human insulin receptor (IR) or transferrin receptor (TfR) [8,12,47]

## Data Availability

The data presented in this review are available and can be found in the cited references.
